# Assessment of soil quality for guided fertilization in 7 barley agro-ecological areas of China

**DOI:** 10.1371/journal.pone.0261638

**Published:** 2022-01-07

**Authors:** Yu Zhou, Yingcheng Fan, Guang Lu, Anyong Zhang, Ting Zhao, Genlou Sun, Daokun Sun, Qi Yu, Xifeng Ren

**Affiliations:** 1 College of Plant Science and Technology, Huazhong Agricultural University, Wuhan, China; 2 Hubei Hongshan Laboratory, Wuhan, China; 3 Biology Department, Saint Mary’s University, Halifax, NS, Canada; Shandong University, CHINA

## Abstract

Soil quality is the basis for the development of sustainable agriculture and may be used for evaluating the sustainability of soil management practices. Soil quality status and integrated soil quality index (SQI) in sampled 97 farmlands distributed in 7 barley agro-ecological areas of China were analyzed by using 13 soil chemical parameters. The results showed six principal components totally explained 72% variability for the 13 parameters and identified 9 parameters (includes pH, NH_4_^+^-N, NO_3_^-^-N, available P, available K, exchangeable Mg, DTPA-Fe, DTPA-Cu and Cl^-^) with high factor loading values as the minimum data set (MDS) for assessing soil quality. Average soil quality of all farmlands is moderate (SQI = 0.62). The SQI of barley farmlands in 7 agro-ecological areas showed the following order: Inner Mongolia Plateau (0.75 ± 0.02) > Yunnan-Kweichow Plateau (0.72 ± 0.06) > Qinghai-Tibet Plateau (0.63 ± 0.08) > Yangtze Plain (0.62 ± 0.10) > Huanghuai Region (0.58 ± 0.09) > Northeast China Plain (0.56 ± 0.07) > Xinjiang Province (0.54 ± 0.07). Total 29 out of 97 farmlands in 7 areas have low SQI level (< 0.55). Hence, these farmlands require urgent attention for soil quality improvement through modification of the soil parameters in the MDS.

## Introduction

Barley (*Hordeum vulgare* L.) is one of the foremost agricultural domesticated crops with wide grown environments of latitude from near the equator to 70°North. It is believed to extend back to 2000 BC in China that barley cultivation probably had originated [1]. Barley grain yield is impeded by genotype × environment × management interactions. High-quality soil is one of the prerequisites for high yield in barley.

Soil quality (SQ) is a comprehensive indicator reflecting the state of soil fertility, which can be interpreted in some features: (1) the potential to function within the managed or natural ecosystems, (2) an ability to keep water and air be conserved, (3) the capacity to sustain plant and animal productivity as well as health [2]. Soil quality is soil- and site-specific, and depends on numerous controlling factors, including physical, chemical and biological properties, and even climate [3, 4]. Several studies acknowledged that soil quality can’t be measured directly [5–7], but soil chemical parameters can be used as proxy indicators of SQ in farmland ecosystem because they are sensitive to changes that is mainly induced by human’s management [8]. Soil pH is often declared the master soil variable since it influences numerous soil chemical, physical and biological properties that affect plant growth [9]. Soil organic carbon (SOC) has a great impact on soil structural stability and productivity of agro-ecosystem [10, 11]. The other important indicators represent soil nutrient status are C, N and P, and their stoichiometries, which can provide information about the balance of elements induced by vegetation growth or succession [12]. Ca^2+^, Mg^2+^, K^+^ and Na^+^ show obvious importance on buffering pH because H^+^ can be substituted by these ions on the cation-exchange site, thereby increases phytoavailability of mineral elements [13]. Appropriate fertilizer-P and S management will guarantee optimal agronomic and economic returns of barley. Meanwhile, adverse environmental impacts will be minimized [14]. Fe, Mn, Cu, Zn, B and Cl are indispensable micronutrients absorbed by plants at a lower level than macronutrients, but the incidence of essential micronutrients deficiency has increased in recent years [15].

Soil quality was commonly investigated in the farmlands planted with corn, rice and wheat [16–19], but rarely done in the farmlands planted with barley. In this study, we investigated 13 soil chemical parameters that greatly contribute to the barley growth in 7 barley agro-ecological areas of China. Based on principal component analysis (PCA), we obtained the minimum data set (MDS) of these parameters and calculated soil quality index (SQI) for assessing soil quality of barley farmlands. Multiple linear regression was performed to explore the factors caused soil chemical parameters significant differences among the areas. The results can provide guidelines to improve soil quality for barley farmland.

## Materials and methods

### Study areas

**Fig 1:**
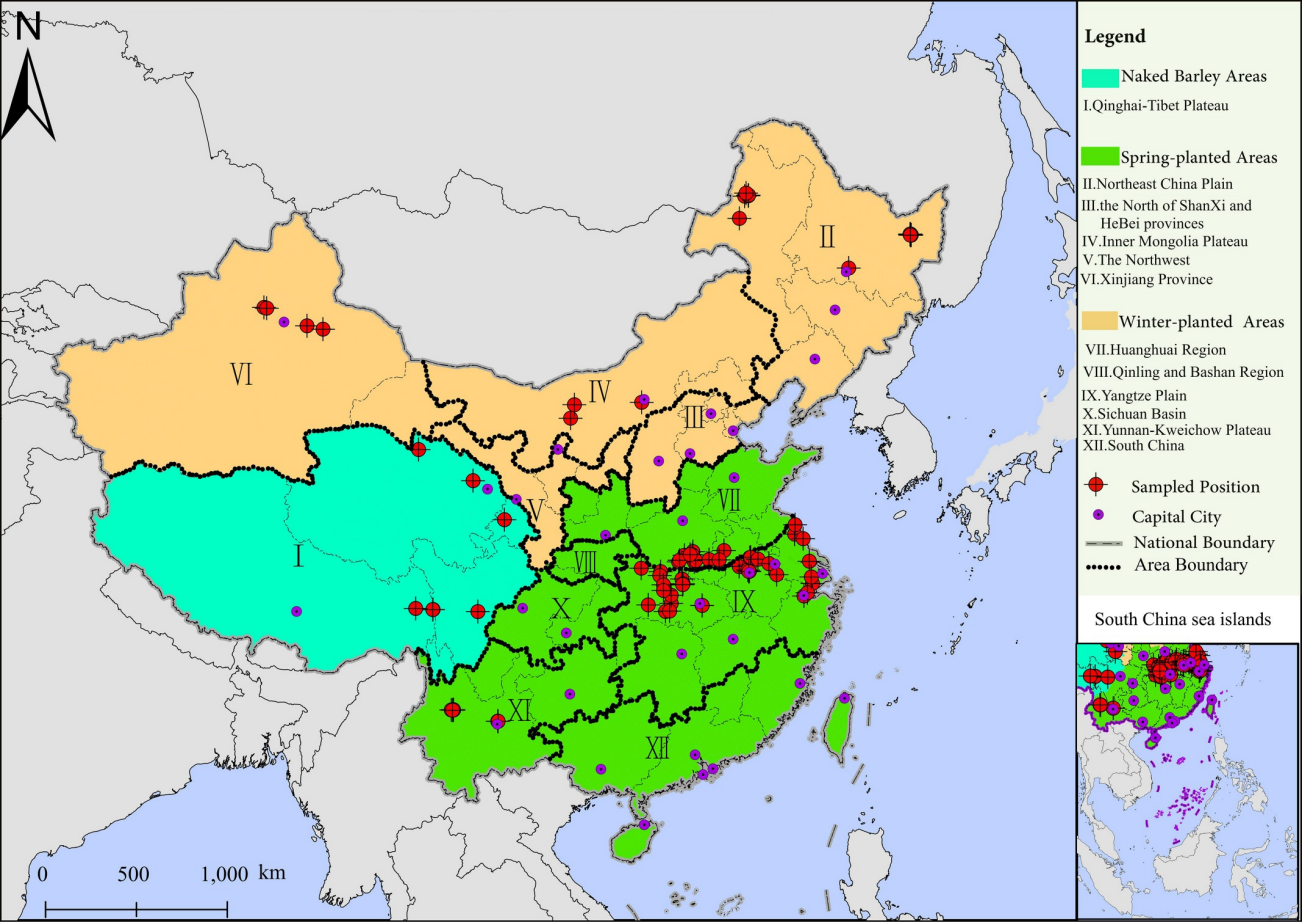
Map of 97 sampled farmlands (location is indicated in a red circle with a coordinate) distributed in 7 of 12 barley ago-ecological areas. The original map was obtained from Natural Earth (http://www.naturalearthdata.com/) and was further processed using software Arcgis 10.2 version.

Based on types of barley grown, barley growth areas in China were classified into Naked barley area, Spring-planted barley area and Winter-planted barley area, respectively, which are further divided into 12 barley agro-ecological areas (Fig 1). Seven out of 12 barley ago-ecological areas were studied including Qinghai-Tibet Plateau, Northeast China Plain, Inner Mongolia Plateau, Xinjiang Province, Huanghuai Region, Yangtze Plain and Yunnan-Kweichow Plateau (Fig 1, S1 Table).

Barley agro-ecological areas in China were divided mainly based on differences in uses of barley products, cropping system, adaptability of varieties, and actual sowing date. Naked barley area (Qinghai-Tibet Plateau and its vicinity) is one of the centers of domestication of cultivated barley where naked barley (Chinese called “Qingke”) is mainly planted and has become staple food of people inhabited in the area [20]. However, the cultivation of barley in Qinghai-Tibet Plateau is severely limited by cold weather, inconvenient transportation and unenlightened cultivation techniques. Spring-planted barley areas (Northeast China Plain, the North of ShanXi and HeBei Provinces, Xinjiang Province, Inner Mongolia Plateau and the Northwest China) account for 70% amount of China’s barley for beer use due to excellent natural conditions (Fig 1). However, the lack of knowledge of soil fertility and cultivation methods for smallholder farmers has hampered yields of high-potential-yield barley varieties. Winter-planted barley areas (Huanghuai Region, Qinling and Bashan Region, Yangtze Plain, Sichuan Basin, Yunnan-Kweichow Plateau and South China) are important feed barley growing areas excluding Huanghuai Region (mainly beer barley) (Fig 1). Since barley has a growth period earlier than wheat, it is easier to achieve high yields of successive crops under one-year-two-harvest cropping system in Winter-planted barley areas. So maintenance of soil quality is an important topic in Winter-planted barley areas under high-intensity planting activities.

### Sampling

A total of 97 farmlands from 7 barley agro-ecological areas were sampled. The number of samples in Qinghai-Tibet Plateau, Northeast China Plain, Inner Mongolia Plateau, Xinjiang Province, Huanghuai Region, Yangtze Plain and Yunnan-Kweichow Plateau were 12, 16, 3, 4, 12, 37, and 13, respectively (Fig 1). The field sites access and samples taking were permitted by farmland holders. In our study, the following criteria were used for the selection of sampling sites and time: (1) All sampled farmlands should be kept away from roads, houses, ditches, dung-hills and fertilizer places; (2) 5 samples were taken from each sampled farmland in a cross five-point sampling method within 25 square meters of the center of the farmland; (3) Soil samples were obtained from 0 to 20cm topsoil at each site on barley developmental stage of the young spike differentiation (a critical period of fertilizer demand of barley) in 2017. Samples from Northeast Plain, Inner Mongolia Plateau and Xinjiang Province were taken in July, Qinghai Tibet Plateau in August, while remaining three areas were sampled in April. (4) After the samples were air dried, the plant residues and stones were removed, and then the 5 samples at each sampling farmland were sieved (< 2mm sieve) and fully mixed.

### Determination of soil chemical parameters

All chemical analyses were conducted by using air-dried soil fractions in College of Plant Science and Technology, Huazhong Agricultural University. pH was measured potentiometrically with a soil/water ratio of 1:1. Soil organic carbon (SOC) were determined according to the chromic acid digestion combined with spectrophotometric procedure [21]. NH_4_^+^-N and NO_3_^-^-N were determined by Nessler colorimetry method and Nitro Indicator by Colorimetric method [22], respectively. Available P was measured by Molybdenum blue colorimetry method [23, 24], and available K by Turbidimetric ultramicro titration [25]. Exchangeable Ca and exchangeable Mg were determined by Ethylene Diamine Tetraacetic Acid (EDTA) titrations [26]. Concentrations of SO_4_^2-^, available B, DTPA-Fe, DTPA-Cu and Cl^-^ were determined by Barium sulfate turbidimetry [27], Colorimetric o-Phenanthroline method [28], sequential extraction method of 0.1M HCl reagent [29], azomethine-H method [30] and Silver chloride turbidimetry [31], respectively.

### Selection of the minimum data set (MDS) and calculation of soil quality index (SQI)

Principal component analysis (PCA) can transform a large number of variables into sets of variables while retaining the information of the original variables as much as possible [32]. Principal components with larger eigenvalue were selected because of their elevated degree of explanation for variability [32]. Under each identified principal component (PC), soil chemical parameters with high factor loadings which were defined absolute values within 10% of the highest factor loading were chosen for MDS [32, 33]. If several soil chemical parameters with high factor loading values appear in the same PC, then a correlation analysis needs to be done to filter highly collinear soil chemical parameters out of the MDS [33]. After the MDS is defined, the weighting factor (*Wi*) for soil chemical parameter is measured by the explained variability of the PC which include the parameters and the cumulative variability of all selected PCs [33]. The formula is as follows:

$$Wi={Vi}_{}/\sum_{1}^{n}Vi_{}$$ (1)

Where *Vi* is the variability explained by the i-*th* principal component, n is the number of selected principal component.

Parameters in the MDS were ranked in all sampled farmlands by linear scoring techniques [33, 34]. The score (*Si*), ranging from 0 to 1, was assigned according to “more is better”, “less is better” or “optimum” rules. The “more is better” rule is appropriate for SOC, NH_4_^+^-N, NO_3_^-^-N, available P, available k, exchangeable Ca, exchangeable Mg and SO_4_^2-^ which are macronutrients and have a favorable impact on soil quality when they exhibit higher concentration, while the “optimum” rule is suitable for pH, available B, DTPA-Fe, DTPA-Cu and Cl^-^ which are micronutrients (except for pH) and have a positive influence up to a certain level beyond which the influence may be considered harmful [33].

SQI was calculated using the following formula:

$$SQI=\sum_{i=1}^{n}Wi*Si$$ (2)

Where *Wi* is the PCA weighting factor of the soil chemical parameters in MDS. *Si* is the corresponding score.

### Effects of natural factors on soil chemical parameters

After multiple comparisons, soil chemical parameters in MDS exhibited significant difference between areas were selected as response variables into multiple linear regression, while natural factors (including elevation, annual average temperature, annual average humidity, average annual rainfall and peak sunshine hours) as explanatory variables. If data in soil chemical parameters did not conform to a normal distribution after Kolmogorov-Smirnov (K-S) test, data were logarithmically transformed. Data of annual average temperature, annual average humidity, average annual rainfall and peak sunshine hours in year 2017 were used, because soil was sampled in that year. These data were obtained from the National Meterological Information Center of China.

### Statistical analyses

The non-parametric Kruskal-Wallis H test was performed in evaluating the differences between areas on all determined soil chemical parameters using SPSS version 22.0. Principal Component Analysis (PCA) was performed using the psych package in R version 3.6.0 for selecting parameters in the minimum data set (MDS). Pearson’s correlations were performed using SPSS version 22.0 in order to filter soil chemical parameters that performed highly collinear with others (the criterion of the correlation coefficient > 0.7) [33]. *Wi*, *Si* and SQI were calculated by Excel, and were shown by TBtools v1.045 [35].

## Results

### Soil chemical parameters of sampled farmlands in 7 agro-ecological areas

**Fig 2:**
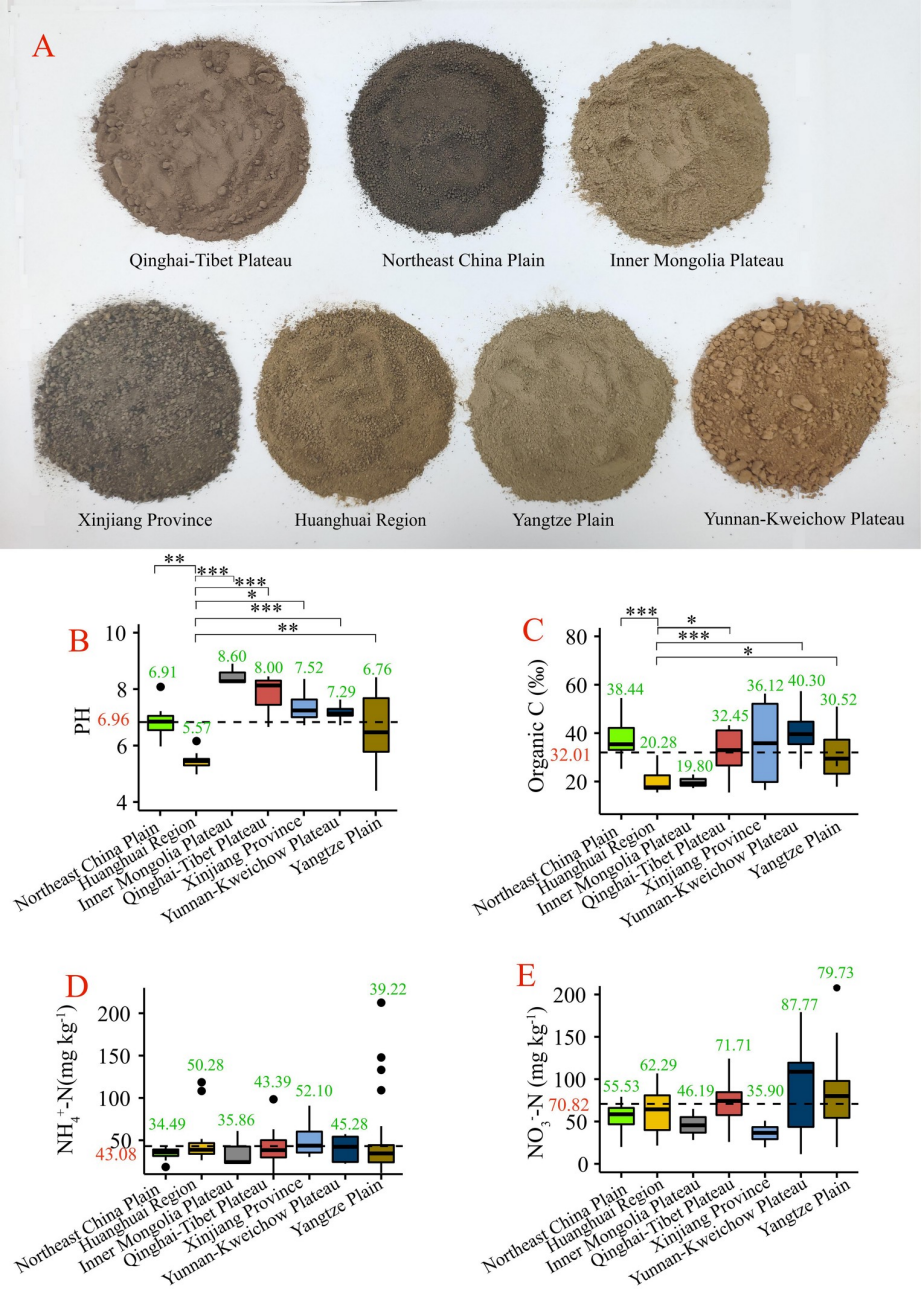
Soil properties of 7 barley ago-ecological areas on colors, pH, soil organic C, NH_4_^+^-N and NO_3_^-^-N. (**A**) Photo of topsoil colors. (**B-E**) difference comparison between farmlands in 7 barley ago-ecological areas on pH, soil organic C, NH_4_^+^-N and NO_3_^-^-N respectively. Data were presented as box plots where boxes represent the inter-quartile ranges. The lines across the boxes, the whiskers and the black circles represented the median values, the 10th and 90th percentiles and outliers, respectively. The black dashed line represented the average levels of all samples. The number above the box represented the average level of parameter in each area. *, ** and *** denoted significant differences by Kruskal-Wallis H test in 0.1, 0.05 and 0.01 levels, respectively.

The color’s investigations of topsoil (0–20 cm) at Qinghai-Tibet Plateau, Northeast China Plain, Inner Mongolia Plateau, Xinjiang Province, Huanghuai Region, Yangtze Plain and Yunnan-Kweichow Plateau revealed large difference between them (Fig 2A). According to pH (Table 1), sampled farmlands were weak acidic in Huanghuai Region (average pH = 5.57), neutral in Northeast China Plain, Yunnan-Kweichow Plateau and Yangtze Plain with the average levels of 6.91, 7.29 and 6.76, respectively (Fig 2B, Table 1). However, the remained three areas showed alkaline soil conditions (Fig 2B, Table 1). Average concentrations of SOC in Yunnan-Kweichow Plateau was the highest (40.30 g kg^-1^), followed by Northeast China Plain (38.44 g kg^-1^), Xinjiang Province (36.12 g kg^-1^), Qinghai-Tibet Plateau (32.45 g kg^-1^), Yangtze Plain (30.52 g kg^-1^) and Huanghuai Region (20.28 g kg^-1^) (Fig 2C). Both NH_4_^+^-N and NO_3_^-^-N showed no difference when each pairwise comparison was performed in two of the seven groups (Fig 2D and 2E).

**Table 1:** The classifications standards for some soil chemical parameters based on the second national soil census and other previous studies.

Soil chemical parameters	*RANK* ^a^						Critical level	Optimal level
	*I*	*Ⅱ*	*Ⅲ*	*Ⅳ*	*Ⅴ*	*Ⅵ*		
pH	< 4.5(Strong acid)	4.5–5.5 (Acid)	5.5–6.5(Weak acid)	6.5–7.5(Neutral)	>7.5(Alkaline)	--	--	6.5–7.8
Available P	>40	20~40	10~20	5~10	3~5	< 3	20–40	--
Available k	>200	150~200	100~150	50~100	30~50	< 30	80–120	--
Exchangeable Ca	>1.0	0.7–1.0	0.5–0.7	0.3–0.5	<0.3	--	--	--
Exchangeable Mg	>0.3	0.2–0.3	0.1–0.2	0.05–0.1	<0.05	--	0.049–0.12	--
SO_4_^2-^	>30	16–30	<16	--	--	--	--	30–50
Available B	>2.00	1.0–2.0	0.5–1.0	0.2–0.5	≤0.2	--	0.3–1.0	2.0
DTPA—Fe	>20	10.0–20	4.5–10	2.5–4.5	<2.5	--	5	>10
DTPA—Cu	>1.80	1.0–1.8	0.2~1.0	0.1~0.2	<0.1	--	1.6–2.0	>6

^a^Classification was recommended by Second State Soil Survey of China (SSSSC) [36].

The critical level and optimal level refer to Paltridge et al. [37], Peverill et al. [38] and references therein, and Zou et al. [15].

**Fig 3:**
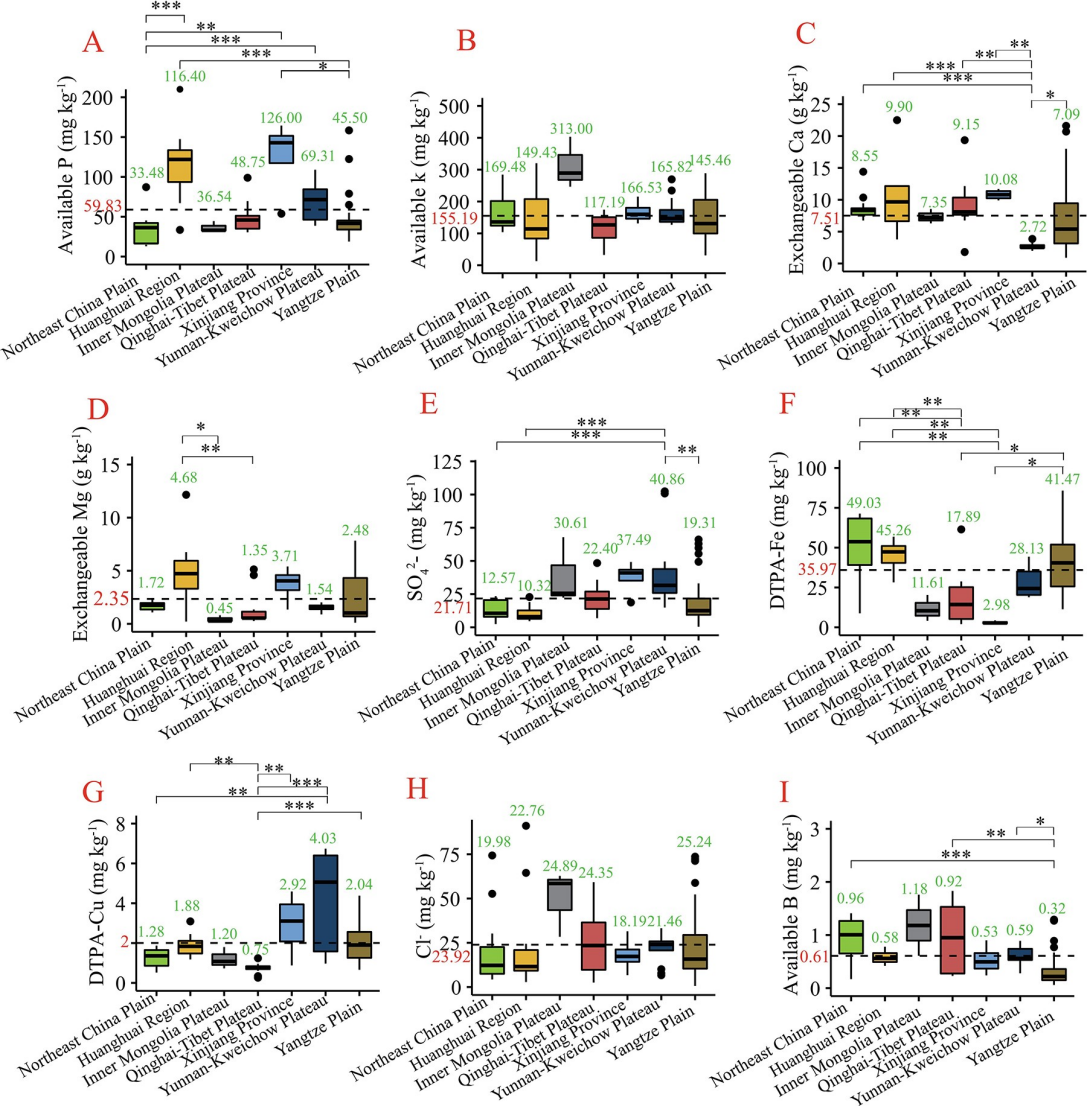
Difference comparison between sampled barley farmlands of 7 barley ago-ecological areas on available P, available k, exchangeable Ca, exchangeable Mg, SO_4_^2-^, DTPA-Fe, DTPA-Cu, Cl^-^ and available B, respectively. Data of each soil chemical parameters were presented as box plots (From A to I) where boxes represented the inter-quartile ranges. The lines across the boxes, the whiskers and the black circles represented the median values, the 10th and 90th percentiles and outliers, respectively. The black dashed line represented the average levels of all samples. The number above the box represented the average level of parameter in each area. *, ** and *** denoted significant differences by Kruskal-Wallis H test in 0.1, 0.05 and 0.01 levels, respectively.

Average available P concentrations in Northeast China Plain (33.48 mg kg^-1^), Inner Mongolia Plateau (36.54 mg kg^-1^), Qinghai-Tibet Plateau (48.75 mg kg^-1^) and Yangtze Plain (45.50 mg kg^-1^) were lower than that in other three agro-ecological areas (Fig 3A). But the differences were not significant (Fig 3A). The sufficient soil K status in Inner Mongolia Plateau averaged 313.00 mg kg^-1^ which much higher than the critical level of 80–120 mg kg^-1^, while critical status of 117.19 mg kg^-1^ was discovered in Qinghai-Tibet Plateau (Fig 3B, Table 1). All of 7 areas had higher average exchanged Ca concentrations than exchanged Mg in each area, suggesting that Ca is the dominant cation (Fig 3C and 3D). Farmlands in both Northeast Plain (average SO_4_^2-^ content of 12.57 mg kg^-1^) and Huanghuai region (average SO_4_^2-^ content of 10.32 mg kg^-1^) exhibited S concentrations at the third level (16 mg kg^-1^) according to classification of SSSSC (Fig 3E, Table 1).

DTPA-Fe, DTPA-Cu, Cl^-^ and available B are indispensable micronutrient for plants growth. Concentrations of DTPA-Fe in all sampled farmlands from Xinjiang Province (2.98 mg kg^-1^) were inadequate, which is much lower than critical nutrient values of 5 mg kg^-1^, while plentiful in Northeast China Plain (49.03 mg kg^-1^), Huanghuai Region (45.26 mg kg^-1^) and Yangtze Plain (41.47 mg kg^-1^) (Fig 3F, Table 1). DTPA-Cu in Northeast China Plain (1.28 mg kg^-1^), Inner Mongolia Plateau (1.20 mg kg^-1^) and Qinghai-Tibet Platea (0.75 mg kg^-1^) were deficient (critical level of 1.6–2.0 mg kg^-1^) (Fig 3G, Table 1). The highest average level of Cl^-^ was found in Yangtze Plain (25.24 mg kg^-1^), lowest in Xinjiang Province (18.19 mg kg^-1^) (Fig 3H). Most areas showed B shortage due to the available B under the critical level of 1 mg kg^-1^ (Fig 3I, Table 1).

### Soil quality index (SQI) of sampled farmlands in 7 agro-ecological areas

The PCA was performed for 13 soil chemical parameters, and identified 6 PCs with larger eigenvalues, which accounting for 14%, 14%, 13%, 11%, 10% and 10% variability from PC1 to PC6, respectively. Overall, 72% of the total variability were explained (Table 2). PC1 was strongly represented by pH (0.89) and DTPA-Fe (-0.84) due to their higher absolute weighted factor loading values. Available P (0.76) and exchangeable Mg (0.77) had high positive loading values on PC2 (Table 2). PC3 was strongly represented only by DTPA-Cu (0.89). NH_4_^+^-N (0.78) and available K (0.74) made up most of PC4, while NO_3_^-^-N (0.87) and Cl^-^ (0.77) dominated PC5 and PC6, respectively (Table 2). These higher weighted soil chemical parameters were selected into minimum data set (MDS).

**Table 2:** Selected principal component in measured soil chemical parameters.

Principal component	PC1	PC2	PC3	PC4	PC5	PC6
Variability(%)	14	14	13	11	10	10
Cumulative(%)	14	28	41	52	62	72
Weighting factor	0.19	0.19	0.18	0.15	0.14	0.14
Eigenvectors loading						
pH	**0.89**	-0.21	0.09	-0.09	-0.01	0.19
Soil Organic C	0.00	-0.33	0.60	-0.07	-0.05	0.32
NH_4_^+^-N	-0.01	0.05	-0.17	**0.78**	0.07	-0.17
NO_3_^-^-N	-0.20	-0.07	-0.07	-0.05	**0.87**	0.62
Available P	-0.10	**0.76**	0.04	0.16	0.22	-0.06
Available K	0.06	-0.09	0.19	**0.74**	-0.10	0.12
Exchangeable Ca	0.42	0.54	-0.35	-0.29	-0.26	0.06
Exchangeable Mg	-0.01	**0.77**	0.08	-0.20	-0.38	0.01
SO_4_^2-^	0.35	0.12	0.61	0.04	0.50	-0.06
Available B	0.11	-0.25	0.11	-0.23	-0.09	0.67
DTPA—Fe	**-0.84**	-0.10	-0.04	-0.15	0.14	0.01
DTPA—Cu	-0.06	0.15	**0.85**	0.04	-0.12	-0.16
Cl^-^	0.04	0.17	-0.13	0.14	0.08	**0.77**

Bold factor-loadings were considered high weighted.

Several soil chemical parameters in MDS were significantly correlated (Table 3). The significant positive correlation occurred between available K and NH_4_^+^-N (r = 0.433; *p < 0*.*01*), so did between DTPA-Fe and NO_3_^-^-N (r = 0.289; *p < 0*.*01*) as well as between exchangeable Mg and available P (r = 0.346; *p < 0*.*01*) (Table 3). On the other hand, the significant negative correlation was discovered between pH and available P (r = -0.295; *p < 0*.*01*) as well as between pH and DTPA-Fe (r = -0.661; *p < 0*.*01*) (Table 3). However, no parameter from MDS was considered redundant because of low correlation coefficient (r < 0.7) between any two parameters (Table 3).

**Table 3:** Pearson correlation coefficient among soil chemical parameters in minimum data set (MDS).

	NH_4_^+^-N	NO_3_^-^-N	Available P	Available K	pH	Exchangeable Mg	DTPA-Fe	DTPA-Cu	Cl^-^
NH_4_^+^-N	1								
NO_3_^-^-N	-0.004	1							
Available P	0.029	0.098	1						
Available K	0.433**	-0.013	0.007	1					
pH	0.023	-0.138	-0.295**	0.048	1				
Exchangeable Mg	-0.139	-0.259*	0.346**	-0.076	-0.062	1			
DTPA—Fe	-0.126	0.289**	-0.127	-0.102	-0.661**	-0.022	1		
DTPA—Cu	-0.098	-0.165	0.081	0.066	-0.058	0.149	0.035	1	
Cl^-^	0.103	-0.063	-0.049	0.033	0.173	-0.035	-0.097	-0.074	1

* and ** denoted significant level in 0.05, 0.01, respectively.

**Fig 4:**
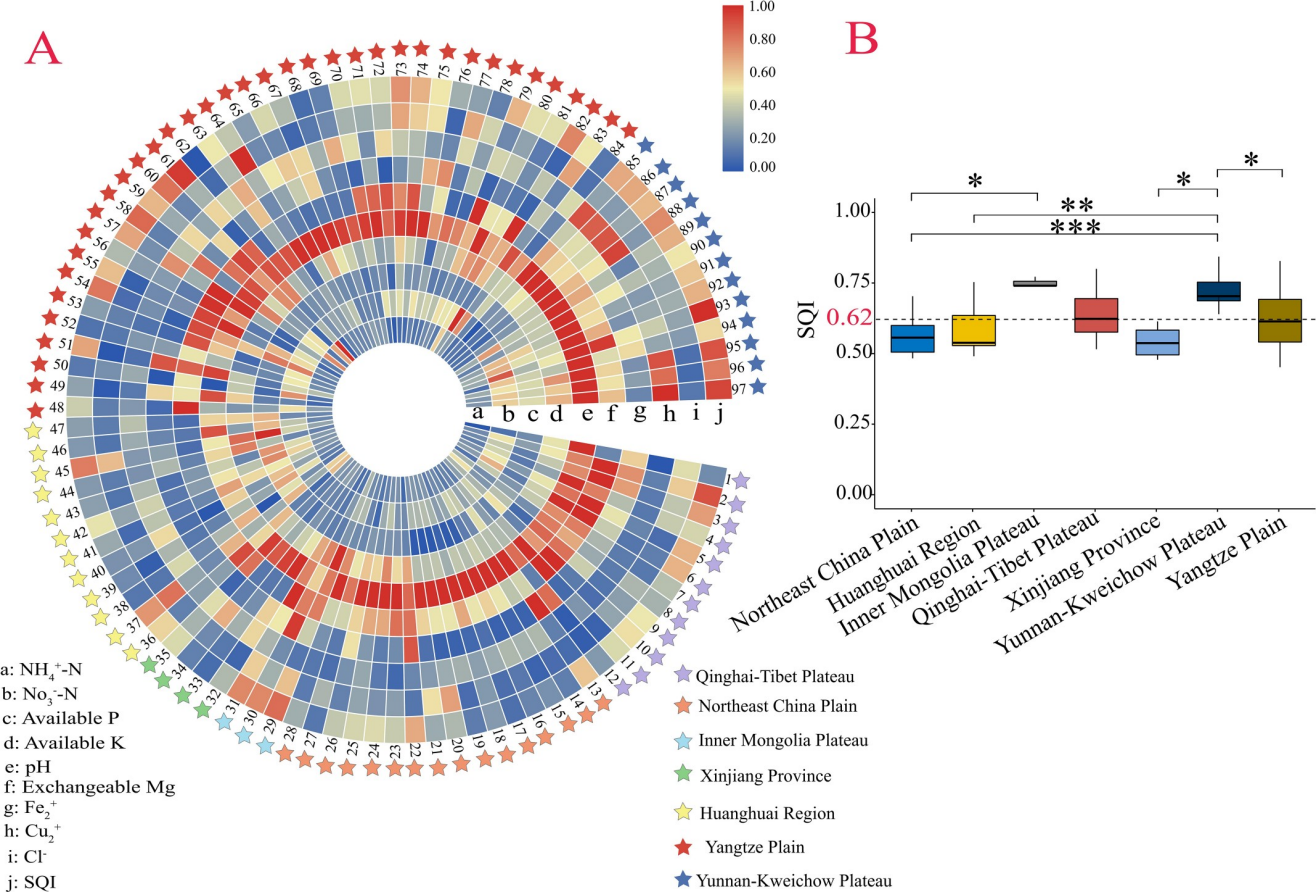
Soil quality index (SQI) on each farmland and difference comparison on 7 barley ago-ecological areas. (**A**) Scores for each soil chemical parameter from Minimum data set (MDS) in 97 sampled farmlands and deriving SQI (data were presented by 0–1 normalization); (**B**) Difference comparison between farmlands of 7 sampled barley ago-ecological areas on SQI. Data were presented as box plots where boxes represented the inter-quartile ranges. The lines across the boxes, the whiskers and the black circles represented the median values, the 10th and 90th percentiles and outliers, respectively. The black dashed line represented the average levels of all samples. *, ** and *** denoted significant differences by Kruskal-Wallis H test in 0.1, 0.05 and 0.01 levels, respectively.

In 97 sampled farmlands, the score (*Si*) of each soil chemical parameter from MDS and the soil quality index (SQI) were presented in Fig 4A. The level of *Si* represents whether the content of soil chemical parameter was suitable for barley growth. Lower pH scores in Huanghuai Region (farmlands from 36 to 47) highlighted adverse soil pH for barley growth (Fig 4A). Adverse soil DTPA-Fe condition for barley growth mainly occurred on most farmlands in Northeast China Plain (farmlands from 14 to 28), Huanghuai Region (farmlands from 36 to 47) as well as part in Yangtze Plain (farmlands from 48 to 84 except for 49, 51, 58, 60, 63, 66, 67, 71, 75, 76 and 78) (Fig 4A). Most farmlands in all areas possessed average or unfavorable Cu status except some farmlands in Yunnan-Kweichow Plateau (farmlands from 85 to 89 and 95 to 97) (Fig 4A).

According to the classifications standards of Marzaioli et al. [39], the SQI was divided into three grades: SQI < 0.55, 0.55 < SQI < 0.70, and SQI > 0.70 was regarded as low soil quality, intermediate soil quality, and high soil quality, respectively. The average SQI (0.62) of all sampled farmlands indicated intermediate soil quality level for barley growth (Fig 4B). Farmlands in Inner Mongolia Plateau had SQI ranging from 0.74 to 0.77 with the highest average SQI of 0.75 ± 0.02, followed by farmlands in Yunnan-Kweichow Plateau (0.72 ± 0.06), Qinghai-Tibet Plateau (0.63 ± 0.08), Yangtze Plain (0.62 ± 0.10), Huanghuai Region (0.58 ± 0.09), Northeast China Plain (0.56 ± 0.07) and Xinjiang Province (0.54 ± 0.07) (Table 4). It’s worth concerning that total 29 farmlands in 7 areas had low SQI level (< 0.55) (Table 4).

**Table 4:** Descriptive statistics of SQI in 7 ago-ecological areas.

Areas	Mean	Maximum	Minumum	Range	SD	CV(%)	Low	Intermediate	High
Northeast China Plain	0.56	0.70	0.48	0.22	0.07	11.68	7	9	0
Huanghuai Region	0.58	0.75	0.49	0.26	0.09	15.07	7	4	1
Inner Mongolia Plateau	0.75	0.77	0.74	0.03	0.02	2.46	0	2	1
Qinghai-Tibet Plateau	0.63	0.80	0.51	0.28	0.08	13.13	2	9	1
Xinjiang Province	0.54	0.61	0.48	0.07	0.06	11.62	2	2	0
Yunnan-Kweichow Plateau	0.72	0.84	0.64	0.20	0.06	8.68	0	9	4
Yangtze Plain	0.62	0.83	0.45	0.38	0.10	15.62	11	22	4

Low: Number of farmlands with SQI < 0.55; Intermediate: Number of farmlands with 0.55< SQI < 0.70; High: Number of farmlands with SQI > 0.70. The classification standards according to Marzaioli et al. [39].

### Soil chemical parameters were influenced by natural factors

In MDS, pH, available P, DTPA-Fe and DTPA-Cu exhibited significant differences among several areas (Figs 2B, 3A, 3F and 3G). Therefore, they were selected as response variables, while natural factors (include elevation, annual average temperature, average annual rainfall, annual average humidity and peak sunshine hours) were collected as explanatory variables to explore the explanation of variation by regression analysis.

The regression analyses showed that variations of pH, available P, DTPA-Fe and DTPA-Cu that were totally explained by natural factors were different (Table 5). Higher R_adj_^2^ of 0.3912 in DTPA-Fe model indicated DTPA-Fe was more easily influenced by these nature factors, followed by pH model (R_adj_^2^ = 0.3297), DTPA-Cu model (R_adj_^2^ = 0.2019) and available P model (R_adj_^2^ = 0.1677) (Table 5).

**Table 5:** Results of regression analyses of parameters from MDS with significant differences in 7 areas.

Soil chemical parameters	R^2^	R_adj_^2^	P value
pH	0.3697	0.3297	<0.001
Available P	0.2111	0.1677	<0.001
DTPA—Fe	0.4229	0.3912	<0.001
DTPA—Cu	0.2434	0.2019	<0.001

R_adj_^2^: variation of parameter was total explained by altitude, annual average temperature, average annual rainfall, annual average humidity and peak sunshine hours.

## Discussion

### Soil quality status of sampled farmlands in 7 agro-ecological areas

Barley is one of the oldest cereal crops which had domesticated more than 5000 years in China, and is mainly used for animal feeding, human food, and malt for brewing [40]. High quality soil is one of the prerequisites for high yield performance in barley. The variable pH, NH_4_^+^-N, NO_3_^-^-N, available P, available K, exchangeable Mg, DTPA-Cu, DTPA-Fe and Cl^-^ retained in MDS for estimating SQI suggested the importance of these soil parameters in maintaining soil quality.

Although soil in Northeast China Plain exhibited neutral pH status (average level of 6.91) (Fig 2B), our research indicated that this area (black soil area) is facing soil acidification, which supported traditional theory that soils are “rich” in K in this area (Fig 3B) [41]. Average NH_4_^+^-N, NO_3_^-^-N and available P are below average concentrations in all farmlands suggested moderate to inferior soil fertility conditions in Northeast China Plain.

Weakly acidic soil in Huanghuai Region was found in our study, which is the opposite of Huang et al. [42], who reported alkaline soil status in this area. This is due to both sampled sites and time are different. It’s worth noting that when soil pH drops below 5, it is likely that crops exhibit reduced water and nutrition uptake due to occurrence on toxicity of aluminum [43]. We agreed that one of the reasons causing soil acidification was excess nitrogen (N) fertilization input [42, 44, 45]. The observed high variability in available P, available K and exchangeable Mg in the studied farmlands within this area indicated great differences in the concentration of these nutrients from one farmland to the other even in the same area (Fig 3A, 3B and 3D). Critical levels of extractable K were suggested to be 78 to 117mg kg^-1^ for most cereal crops, and possibly around 100 mg kg^-1^ on heavier textured soils [38]. This indicated K was marginal or deficient on some farmlands of this area (Fig 3B). Sufficiency in DTPA-Fe (> 5 mg kg^-1^ critical level) and non-toxicity in Cl^-^, while margin of inadequacy in DTPA-Cu (1.6–2 mg kg^-1^ critical level) suggested Cu might be a limiting factor for soil quality in this area (Fig 3F and 3G) [15].

Our result on alkaline soil status in sampled farmlands from Inner Mongolia Plateau is consistent with Yang et al. [46]. Lower NH_4_^+^-N, NO_3_^-^-N and available P, exchangeable Mg, and DTPA-Cu pointed out that these parameters are limiting factors for soil quality in this area, especially DTPA-Cu (< 1.6–2 mg kg^-1^ critical level) (Figs 2D, 2E, 3A, 3D and 3G). Cu availability could be reduced in slightly basic pH conditions of soil due to soil chemical components favorably binding Cu above soil pH 7.0 [47]. However, soil was sufficient in available K, DTPA-Fe and Cl^-^ (Fig 3B, 3F and 3H).

Results on soil status in Qinghai-Tibet Plateau were similar to the results of Paltridge et al. [37], which soil performed both alkaline and low K status. Furthermore, two mineral elements K and Mg were found to had low contents in the soil samples we tested (Fig 3B and 3D), which favors that third to one half of cereal crops (barley and wheat) were marginal or deficient for K, and Mg [37]. Some farmlands have been discovered inadequate supplement of Fe, while all farmlands have shown a lack of Cu in this area (Fig 3F and 3G). Hence, K, Mg, Fe and Cu might be limiting factors for soil quality in this area.

The pH, available K and DTPA-Cu levels in farmlands of North Xinjiang were similar to the result of Lyu et al. [48]. NO_3_^-^-N, DTPA-Fe and Cl^-^ in this area showed lower levels compared with other areas in temperate regions, tropical regions or semiarid areas due to the particular background of the soil and climatic conditions (Figs 2E, 3F and 3H). Insufficient DTPA-Fe (< Critical level of 5mg kg^-1^) in sampled farmlands from Xinjiang Province might be caused by alkaline soil that is imparted by bicarbonate (Fig 3F) [49].

Karst landforms are the major natural landscapes in Yunnan-Kweichow Plateau. Soil parameters in this area are influenced easily by many factors, such as soil parent material, rock exposure rate, land use and slope position [50]. Soil pH (7.29), available K (165.82 mg kg ^-1^) and DTPA-Cu (4.03 mg kg ^-1^) in this area were approximative with the data from Second State Soil Survey of China (SSSSC) (Figs 2B, 3B and 3G) [36]. No any soil nutrient below critical level in this area indicated high soil quality for ensuring barley’s normal growth.

The neutral soil status was discovered in Yangtze Plain (Fig 2B), but some farmlands exhibited severe acidified status that might impede absorption of other nutrients. According to the classification of the SSSSC, an NH_4_^+^-N content > 90 mg kg^−1^ is considered as medium to high content [51]. NH_4_^+^-N content in this area is under the sufficient level (Fig 2D), which might be related to more fertilizers in such high intensity planting system. A report indicated that NH_4_^+^-N is no longer a restricted parameter in the farmlands in southern China [52]. According to the description of SSSSC [36] and Chen et al. [51], our results suggested that available P and available K were insufficient in this area (Fig 3A and 3B). Over critical levels of exchangeable Mg, DTPA-Fe, DTPA-Cu, and Cl^-^ suggested that none of these parameters play a role in hindering barley growth (Fig 3D and 3F–3H, Table 1).

In addition, barley grown in different areas had different uses. Different fertilizers applied for different type of barley might be resulted in difference in the assessment of soil quality.

### Soil quality assessment and fertilization advice

Soil quality is the basis for the development of sustainable agriculture and it may be used for evaluating and determining the sustainability of soil management practices [53]. In order to assess soil quality, contents of some indicators should be monitored [5, 54], and a single value represented the soil quality should be transformed. Many measured parameters are redundant and highly collinear with others when assessing soil quality, so it is necessary to filter them by principal component analysis combining with correlation analysis, and then generate the minimum data (MDS) that directly determines soil quality index (SQI) [4, 33, 55].

The average SQI in 7 agro-ecological areas is moderate (Fig 4B). No farmlands in Xinjiang exhibited high soil quality (characterized as SQI > 0.70) in this study (Fig 4B). The result is consistent with that in Gong et al. [56]. There are multiple explanations in this low soil quality phenomenon that soil nutrient contents were fast reduced and land degradation occurred during long-term planting in arid regions [57–59]. The relatively high SQI of farmlands in Yunnan-Kweichow Plateau may be attributed to all farmlands in this area with suitable pH condition (6.5~7.8) for barley growth, along with high N content in high percentage of farmlands.

Among the chemical parameters that strongly affect soil quality indicators, variations of pH, DTPA-Fe, DTPA-Cu and available P were totally explained by the nature factors by 32.97%, 39.12%, 20.19% and 16.77% (Table 5), respectively. However, more residual variations were explained by other factors. So, we supposed that the more important contributors to driven variation of soil parameters were parent materials and management activities. It was reported that contents of soil-extractable P on a andesite (igneous rock) site were higher than two sites with different sedimentary rocks in forests of Panama [60]. Compared with granodiorite and schist, the lower vegetation productivity on granite may be partly attributed to the greater proportion of shallow soils and the sandy to loamy sand texture [61]. However, nutrient stress (especially P-availability) was a limited factor by vegetation growth on granite [61]. These reports exhibited great significance for us to understand the relationship between parent material and these soil chemical parameters.

Some suggestions were given for the farmlands with SQI < 5.5. Input of appropriate amounts of lime combining with appropriate restriction of N fertilizer is a solution to improve anthropogenic acidification soil even though it would be arduous for decreasing N fertilizer in China [44]. Some methods to supplement K, such as incorporation of crop residues in the soil or use of cover crops are necessary if insufficient soil K are discovered [47]. Although all farmlands we studied didn’t performed insufficiency of exchangeable Mg (soil critical level of 0.048–0.12 g kg^-1^) [37], Mg deficiency could occur in soils of a low cation-exchange capacity (CEC) [13, 37]. So, the application of proper amount of lime is a supplement approach. When deficiency symptoms of Fe appear on the youngest leaves of barley, foliar application of Ferrous sulfate is an effective way to supplement Fe [62]. In addition, humic substances such as commercial preparations of humic acid or fulvic acid or extracts of peat can supply chelated Fe in soil, which has been used successfully in grasses or dicotyledonous plants [63, 64]. According to the description by Barker and Pilbeam [47], chlorine deficiency in plants is seldom observed in agriculture or nature due to the plentiful supplies in the environment and its redistribution from natural occurrences such as rainfall, marine aerosols, and volcanic emissions. Toxicity of excessive chlorine should be considered although barley is moderately tolerant for it.

## Conclusions

In this study, a soil quality index (SQI) was developed in each sampled farmland to assess soil quality from 7 agro-ecological areas in China. Average soil quality in all farmlands is moderate. Farmlands in Inner Mongolia Plateau exhibited higher SQI, followed by Yunnan-Kweichow Plateau, Qinghai-Tibet Plateau, Yangtze Plain, Huanghuai Region, Northeast China Plain, and Xinjiang Province. Total 29 farmlands in 7 areas were identified with low SQI level (< 0.55). Hence, these farmlands require urgent attention in improving soil quality by modifying soil parameters in Minimum data set (MDS). Our research provides useful guidance for fertilization. It is not only the prerequisite for guaranteeing the high yield of barley, but also for maintaining the sustainability of soil.

## Supporting information

S1 Table Sampling location and 13 soil chemical parameters. (XLS)
